# Directed evolution of human scFvs in DT40 cells

**DOI:** 10.1093/protein/gzv058

**Published:** 2015-10-30

**Authors:** Alfred W.Y. Lim, Gareth T. Williams, Cristina Rada, Julian E. Sale

**Affiliations:** Medical Research Council Laboratory of Molecular Biology, Francis Crick Avenue, Cambridge CB2 0QH, UK

**Keywords:** affinity maturation, *in vitro* selection, monoclonal antibodies, scFv, somatic hypermutation

## Abstract

Cells that constitutively diversify their immunoglobulin genes can be used for selection of novel antibodies and for refining existing affinities and specificities. Here, we report an adaptation of the chicken DT40 system wherein its capacity for somatic hypermutation is harnessed to evolve human antibodies expressed as single-chain variable fragments (scFvs). Expression of membrane-anchored scFvs from within the rearranged Igλ locus created self-diversifying scFv libraries from which we could both select scFvs of a desired specificity and evolve both the specificity and affinity of existing scFvs by iterative expansion and selection. From these scFvs, we were able to create fully human IgG antibodies with nanomolar affinities. We further enhanced the functionality of the system by creating a pool of DT40 scFv lines with high levels of mutation driven by the overexpression of a hyperactive variant of activation-induced deaminase. From this library, we successfully isolated scFvs that bound the spliceosome factor CWC15 and the cytokine human IFNγ. Our results demonstrate the flexibility and utility of DT40 for rapid generation of scFv repertoires and efficient selection, evolution and affinity maturation of scFv specificities.

## Introduction

Monoclonal antibodies are transforming medicine and have become a major sector in therapeutics (Ecker *et al.*, 2015). Their efficacy relies on the identification of molecules that exhibit high specificity and affinity for the desired target. Current human therapeutic antibodies are derived from two broad approaches that either harness *in vivo* B-cell affinity maturation or rely on selection from large display libraries.

Immunisation of human immunoglobulin translocus mice accesses the full power of *in vivo* antibody diversification and selection (Brüggemann *et al.*, 1989; Lonberg *et al.*, 1994; Green *et al.*, 1994). However, it can be limited by immune tolerance preventing the generation of antibodies against conserved mammalian proteins and is expensive. Library display technologies, exemplified by phage display (McCafferty *et al.*, 1990; Breitling *et al.*, 1991; Kang *et al.*, 1991), rely on enormous starting repertoires that can be repeatedly interrogated for the desired specificity. While the latter approach can access epitopes that do not generate good responses in mice, further affinity maturation requires *in vitro* mutagenesis (Gram *et al.*, 1992; Chowdhury and Pastan, 1999; Boder *et al.*, 2000), which can be cumbersome and time-consuming.

Cell lines that constitutively hypermutate their immunoglobulin (Ig) loci (Buerstedde *et al.*, 1990; Kim *et al.*, 1990; Sale and Neuberger, 1998; Sale *et al.*, 2001) provide a potentially attractive alternative to these two approaches. In these B-cell lines, the expression of activation-induced deaminase (AID) drives preferential diversification of the immunoglobulin loci. In the case of human B-cell lines, this diversification takes the form of single base substitutions (somatic hypermutation (SHM)). In the chicken cell line, DT40, Ig diversification proceeds by a combination of SHM and gene conversion, in which tracts of homeologous sequence from upstream pseudogenes are copied into the rearranged Ig genes (Buerstedde *et al.*, 1990; Kim *et al.*, 1990). Preventing gene conversion, either by inhibiting the early steps of homologous recombination, by removing the pseudogenes or by replacing the rearranged Ig genes, results in diversification solely by SHM (Sale *et al.*, 2001; Arakawa *et al.*, 2004, 2008). The feasibility of coupling continuous Ig diversification with *in vitro* selection has been demonstrated for both human cell lines, such as the Burkitt lymphoma line Ramos and the chicken cell line DT40 (Cumbers *et al.*, 2002; Seo *et al.*, 2005; Yabuki *et al.*, 2012). DT40 cells have additional advantages in that they are genetically tractable and can generate higher mutation loads than Ramos, in part due to their very short generation time. DT40 has been used successfully to engineer novel variants of green fluorescent protein with stronger fluorescent intensity (Arakawa *et al.*, 2008) and a ligand trap for Angiopoietin-2 based on mutation and selection of the ectodomain of the receptor tyrosine kinase Tie2 (Brindle *et al.*, 2013).

Here, we report an adaptation of the DT40 system to allow the straightforward selection and evolution of fully human antibodies. We show that human single-chain variable fragments (scFvs) can be efficiently mutated and selected in DT40. We demonstrate the potential of this approach against a series of model antigens and show the feasibility of improving both the starting repertoire of V genes and the mutation rate.

## Materials and methods

### Cell culture conditions

Chicken DT40 B cells were propagated in RPMI-1640 (Gibco/Life Technologies) supplemented with 10% serum (7% fetus bovine serum, 3% chicken serum), 1% Penicillin–Streptomycin and 100 µM β-mercaptoethanol at 37°C with 10% CO_2_. Cells were maintained at a density between 0.5–2.0 × 10^6^ per ml. Human HEK293T and U-2OS cells were propagated in DMEM (Gibco/Life Technologies) supplemented with 10% fetus bovine serum and 1% Penicillin–Streptomycin at 37°C with 10% CO_2_. Cells were maintained at a density between 0.5–3.0 × 10^6^ per plate.

### Plasmids

For targeted insertion of scFvs into the rearranged Igλ locus, the scFv–HEL–TM transgene was cloned into the NheI and BglII sites of the pHypermut2 vector (Arakawa *et al.*, 2008). For transient transfection into HEK293T or U-2OS cells, the scFv of interest was fused with HEL–TM and cloned into pCDNA3.1+ (Life Technologies). For overexpression of human AID variants, FLAG-tagged wild-type human AID and hAID^up^ 7.3 (Wang *et al.*, 2009) was cloned into the EcoRI and NotI sites of the pEAK8 expression vector (Edge Biosystems). For the production of recombinant scFv-Fc, pCDNA 3.1+ was modified to contain a splice acceptor site, hinge, CH2 and CH3 of human IgG1. The scFvs of interest were cloned into this plasmid as a HindIII/BglII fragment in-frame so as to use the splice acceptor to produce an scFv-human Fc protein. For the production of recombinant IgG1, the heavy and light chain V genes from the scFvs of interest were cloned into the human γ1 or human κ expression vectors (Tiller *et al.*, 2008), a kind gift of Hedda Wardemann, as AgeI/XhoI or AgeI/BsiWI fragments, respectively.

### Electroporation of DT40 cells

For targeted integration of scFv into the DT40 Igλ locus, 2 × 10^7^ wild-type DT40 cells were transfected with 30–50 µg of NdeI-linearized pHypermut2-scFv–HEL–TM. Electroporation was performed using a 4-mm electroporation cuvette (Bio-Rad) and a Bio-Rad Gene Pulser set to 550V and 25 µF. Selection with 1.0 μg/ml of puromycin was applied at 24 h and the cells distributed into 96-well plates. For random integration of hAID variants into DT40, 2 × 10^7^ Tomlinson 817 DT40 clones were transfected with 30–50 μg of either the pEAK8 empty vector or the pEAK8-hAID constructs in a 4 mm electroporation cuvette (Bio-Rad). Electroporation was performed using the Bio-Rad Gene Pulser set to 250V and 950 µF. All subsequent steps were similar to the protocol described earlier, with selection at 2 mg/ml G418.

### PCR and Southern blot screening for targeted clones

Genomic DNA was extracted using the Puregene^®^ DNA Isolation Kit (Qiagen). Successful targeting was first verified by PCR using the primer pairs Pλ1 (5′GCCTTGTTGTAGCTTAAATTTTGC) and Pλ2 (5′TTTCCGAAAAAAATGACAAAATATT). Clones shown to be targeted by PCR were confirmed by Southern blot analysis using XcmI digested genomic DNA. The Southern blot probe was amplified from genomic DNA of wild-type DT40 cells using the primer pairs SBP1 (5′ATGTAGTCTGCTGTATGCTT) and SBP2 (5′CTGGCCTGCAAGAGAGAGGCAGA) and then labelled with ^32^P-dCTP using the NEBlot Kit (New England Biolabs).

### DNA sequencing of scFv transgenes

The scFv coding regions were PCR-amplified from genomic DNA of DT40 clones using the appropriate primer pairs. scFvs from the Tomlinson library were PCR-amplified using primer pairs Tom1 (5′CTCGCGGCCCAGCCGGCCATGGCCGAGGTGCAGCTGTTGGAG) and Tom2 (5′CATGCATGTGCGGCCGCCCGTTTGATTTCCACCTTGGTCCCTT) whereas scFvs based on anti-FITC framework were PCR-amplified using primer pairs FITC1 (5′CTCGCGGCCCAGCCGGCCATGGCCCAGGTGCAGCTGGTGGAG) and FITC2 (5′CATGCATGTGCGGCCGCACCTAGGACGGTAAGCTTGGTCCCAGTTC). scFvs based on the anti-DNP framework were PCR-amplified using primer pairs DNP1 (5′GGAGCTGTATCATCCTCTTCTTGGCAGCAACAGCTACAGG) and DNP2 (5′CGCTTCATAGCCGCTGCCAGCTCACATCGTCC). The PCR products were gel purified, cloned into the pCR^®^ Blunt Vector (Life Technologies) and transformed into competent *E. coli* DH5α. Plasmid DNA was extracted from randomly selected bacterial colonies and the scFv sequenced.

### Conjugation of target antigens

Lyophilized casein, keyhole limpet haemocyanin (KLH), thyroglobulin, human IgG, ubiquitin, human serum albumin and mouse IL-33 were purchased from Sigma–Aldrich, UK. Lyophilized human IFNγ was purchased from R&D Systems, UK. Recombinant hCWC15 [aa 80–229] was expressed and purified in our laboratory. Streptavidin–FITC and streptavidin–PE were purchased from Dako Cyomation. Streptavidin–Dylight_650_ was purchased from Thermo Scientific. DNP–BSA–Biotin was purchased from Biosearch Technologies. Biotinylation of antigens was performed using the EZ-Link^®^ Sulfo-NHS-LC-Biotin (Thermo Scientific) according to manufacturer's instructions. Antigen conjugation with Dylight_650_ was performed using the DyLight^®^ Amine-Reactive Dyes (Thermo Scientific) according to manufacturer's instructions. Upon completion of the biotinylation and dye conjugation reactions, the antigens were dialysed in PBS for a total of 4 h at 4°C, with a change of the PBS after the first 2 h. Antigen-coupled paramagnetic beads were made by using biotinylated antigens coupled to M-280 Streptavidin Dynabeads (Life Technologies) according to the manufacturer's instructions.

### Flow cytometry analysis and fluorescent-activated cell sorting

For flow cytometry analysis, up to 4 × 10^6^ DT40, HEK293T or U-2OS cells were harvested each time by centrifugation at 1000 rpm, 4°C for 10 min. For fluorescent-activated cell sorting (FACS), up to 5 × 10^7^ DT40 cells were harvested. The cells were washed once with PBS before staining with the appropriate antibodies and/or antigens. Surface human scFv/HEL expression was detected each time by staining with a mouse anti-HEL F10 antibody (a kind gift of Dr R. Poljak). All antibodies and antigens were diluted in PBS/1% BSA for staining. Antigens at final concentrations of 10–100 nm were used each time. Cells were stained for 20 min on ice, with washings in between each staining step using cold PBS. Stained cells were resuspended in cold PBS/1% BSA and analysed on either the FACS Calibur (Becton Dickinson) or the BD LSRII (Becton Dickinson). Flow cytometry plots were made using FlowJo Version 9. FACS was performed on either the MoFlo High Speed Cell Sorter (Propel Labs) or the Synergy Cell Sorter (Sony Biotechnology).

### Cell sorting with magnetic beads

Up to 1 × 10^9^ DT40 cells were harvested by centrifugation at 1000 rpm, 4°C for 10 min and washed once with cold PBS. Cells were blocked in PBS/1% BSA and rotated at 4°C for 1 h. Cells were then washed once with cold PBS and resuspended in PBS/1% BSA mixed with antigen-coated magnetic beads. The mixture was rotated at 4°C for 1 h after which it was washed with PBS/1% BSA. MACS (Magnetic Activated Cell Sorting) selection was then performed using LD columns (Miltenyi Biotec) on a QuadroMACS Separator (Miltenyi Biotec). Cells were eluted from the LD column using complete DT40 media, and recovered cells were cultured as before. For selection using Dynabeads, 1 × 10^7^ beads were first washed with 1 PBS/1% BSA using a magnetic separator and resuspended with biotinylated antigen at room temp for 30 min. Beads were then washed three times with 1 ml of PBS and resuspended in 50 µl PBS/1% BSA. Up to 1 × 10^8^ cells were washed with PBS and then resuspended in 1 ml 1%BSA/PBS in a 1.5-ml microcentrifuge tube. The antigen-bound beads were added to the cells and rotated for 1 h at 4°C after which a magnetic separator was used to bind the beads to the side of the tube. Bound beads were washed gently twice with 1 ml PBS/1% BSA and resuspended in 100 µl cell culture medium and incubated in a well of a 96-well plate prior to expansion.

### Transfection of HEK293T and U-2OS cells

Transient transfection of HEK293T cells was performed in six-well plates using Genejuice (Novagen) according to the manufacturer's instructions. Transient transfection of human U-2OS cells was performed using the Amaxa^™^ Cell Line Nucleofector^™^ Kit V (Lonza) according to the manufacturer's instructions. Cells were harvested after ∼48 h post-transfection and analysed by flow cytometry for surface scFv/HEL expression and antigen binding.

### Western blotting

For immunoblot analysis of DT40 cell lysates, up to 1 × 10^7^ cells were harvested and resuspended in 200 µl of 1× NuPAGE^®^ LDS Sample Buffer (Life Technologies). The lysate–LDS mixture was denatured at 100°C for 10 min and was resolved on a 4–12% NuPAGE^®^ Bis–Tris polyacrylamide gel (Life Technologies) and then blotted to an Immobilon-P PVDF membrane (Millipore) overnight using the Novex transfer apparatus (Life Technologies). The membrane was subsequently blocked for 1 h at 4°C with 5% milk in PBS/0.1% Tween-20 before probing with either a 1:15 000 dilution of mouse anti-FLAG HRP (Sigma) or 1:10 000 dilution of a goat anti-β-actin antibody (Sigma) in 2.5% milk/PBS/01% Tween-20. The membrane was washed three times with PBS/0.1% Tween-20, and the bound antibody was detected by staining with Amersham™ ECL™ Western Blotting Detection Reagent (GE Healthcare).

### Expression and purification of recombinant IgG1 and scFv-Fc

Two hundred micrograms of plasmid DNA was prepared in a 50 ml solution of Opti-MEM medium (Invitrogen) containing 0.8 µm branched chain polyethylenimine (Sigma) and overlain onto 3 × 10^7^ HEK293T cells in three 175 cm^2^ for 4 h at 32°C. Cells were washed and then incubated for 3 days at 32°C in 150 ml of DMEM (Invitrogen)/0.05% fetus bovine serum. Recombinant antibody protein was harvested from the culture supernatant using protein A sepharose (GE).

### Assembly of an scFv library

Random scFv of unknown binding specificities were PCR-amplified from the Tomlinson synthetic scFv library (de Wildt *et al.*, 2000) using the TL1 (5′CGGCTAGCATGGAGTTTGGGCTGAGCTGGCTTTTTCTTGTGGCTATTTTAAAAGGTGTCCAGTGTGAGGTGCAGCTGTTGG) and TL2 (5′CCCCGTGATGGTGATGATGATGTGCGGCCGC). For CDR3 grafting of the anti-FITC scFv, unique MluI and BamHI restriction sites were first introduced as silent mutations to flank the CDR3 of *V*_H_ of the scFv. This allowed easy exchange of this domain with other CDR3s from a set of human antibodies obtained from humanized translocus mice that were immunized with the HIV-1 antigen gp140 (Pruzina *et al.*, 2011). Novel scFvs obtained in this way were fused to the HEL–TM module as before and cloned into the pHypermut2 expression vector (Arakawa *et al.*, 2008).

### ELISA

ELISAs were performed on Nunc Maxisorb Immunoplates (Thermo Scientific). Antigens were diluted in PBS and adsorbed onto the immunoplates for 2 h at room temperature and blocked with PBS/1% BSA for 30 min at room temperature or overnight at 4°C. The plates were washed four times in PBS after which antibodies, diluted in PBS/1% BSA, were added to the appropriate wells and incubated at room temperature for 2 h. Unbound antibody was washed from the plate with PBS prior to the addition of a secondary antibody coupled to HRP and incubated at room temp for 2 h. After washing off the unbound secondary antibody, the ELISA was developed by the addition of 1 Step Ultra TMB-ELISA substrate solution (Thermo scientific) and the reaction was stopped using 0.5 mM H_2_SO_4_.

## Results

### Efficient hypermutation of human and mouse scFvs in the chicken immunoglobulin light chain locus

**Fig. 1:**
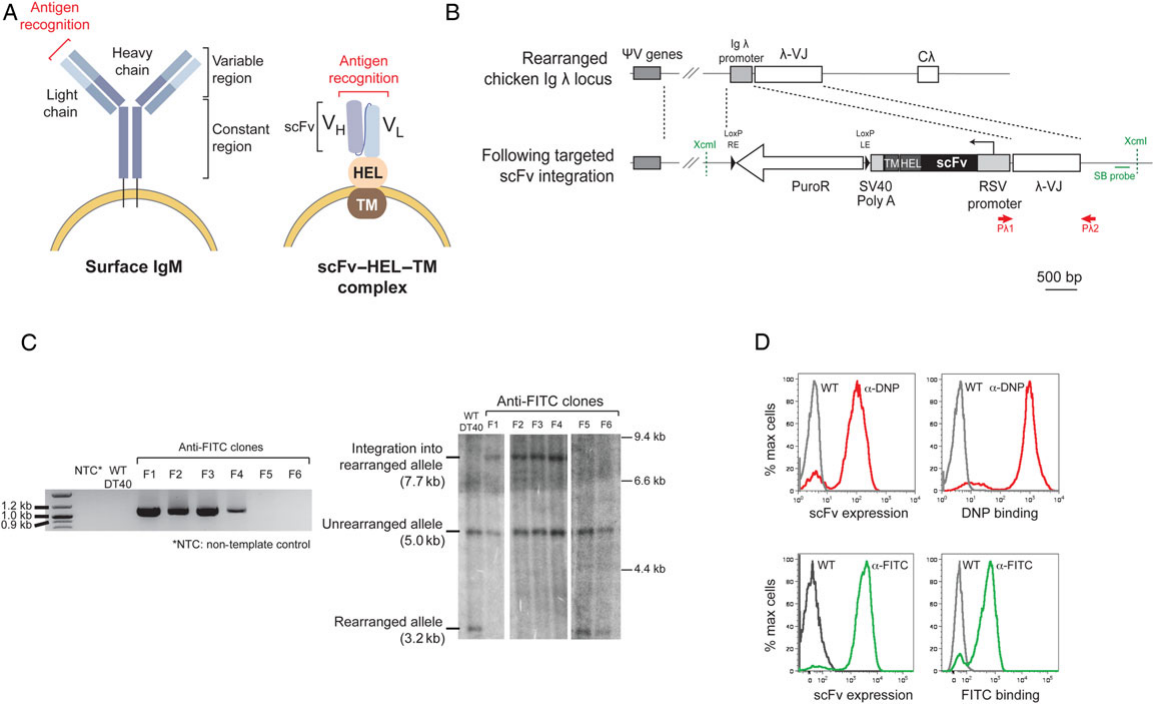
Generation of scFv-expressing DT40 cells. (**A**) Cartoons comparing the structure of the endogenous surface chicken IgM with the expected structure of an scFv–HEL–TM complex. The scFv consists of a *V*_H_ domain joined to a *V*_L_ by a short glycine–serine linker and is fused to a surface expression cassette comprising HEL and an MHC Class I TDTM (HEL–TM). (**B**) Physical map of the rearranged Igλ locus before and after targeted integration. The 5′ homology arm upstream of the chicken Igλ promoter and the 3′ homology arm containing the rearranged Igλ-VJ allele are indicated between the dotted lines. The endogenous chicken Igλ promoter is replaced by the scFv–HEL–TM expression cassette and the puromycin resistance (puroR) cassette in a correctly targeted clone. For screening targeted clones, the positions of the PCR primers Pλ1 and Pλ2 are shown in red and the location of the Southern blot probe (SB probe) and XcmI restriction sites in green. (**C**) *Left:* correctly targeted clones (F1–F4) yield a PCR amplicon of 1055 bp, whereas non-targeted clones (F5 and F6), wild-type DT40 and the non-template control do not. *Right:* on a Southern blot, the probe identifies a 7.7-kb XcmI restriction fragment in correctly targeted clones (F1 to F4), whereas a 3.2-kb fragment is seen in non-targeted clones (wild-type DT40 and clones F5 and F6). (**D**) Histograms depicting surface expression of mouse anti-DNP scFv (top panel) and human anti-FITC scFv (bottom panel) in targeted DT40 clones, detected using an anti-HEL antibody. These surface scFvs remained functionally active and bound their cognate ligands DNP and FITC, respectively.

In order to create surface displayed scFvs in DT40 that are targeted by SHM, we adapted a previously described strategy for knocking-in genes to the immunoglobulin light chain locus (Arakawa *et al.*, 2008). The scFv, in which immunoglobulin *V*_H_ and *V*_L_ segments are joined with a short glycine–serine linker sequence, was fused to a module comprising hen egg lysozyme (HEL) and the MHC Class I transmembrane domain (TM) (Fig. 1A). This construct not only anchors the scFv onto the cell membrane but also allows surface expression to be monitored with antibodies against HEL. To examine the feasibility of this strategy, we initially introduced a human scFv with specificity for the fluorescent dye fluorescein isothiocyanate (FITC) (Vaughan *et al.*, 1996) and a mouse scFv with specificity for the hapten dinitrophenol (DNP) (James *et al.*, 2003). Following transfection and selection of clones, we confirmed targeting into the rearranged allele by Southern blotting and PCR (Fig. 1B and C) since only one of the two DT40 light chain alleles is rearranged and permissive for hypermutation. Both constructs expressed well on the cell surface of DT40 and retained the ability to recognize their canonical antigens (Fig. 1D). The endogenous chicken AID in the DT40 cells targeted both scFvs resulting in their point mutation (Supplementary Fig. S1). The mutagenesis was biased towards the CDRs within the *V*_H_ segment, consistent with the previously described reach of the hypermutating domain downstream of a promoter (Lebecque and Gearhart, 1990; Rada *et al.*, 1994; Rada and Milstein, 2001) and the CDR-biased targeting of AID-mediated SHM in unselected populations (Betz *et al.*, 1993). As expected, there was no evidence of gene conversion given the lack of homology between the scFv and chicken *V*_L_ pseudogenes.

### Convergent evolution of distinct scFvs to recognize the same antigen

*De novo* diversification of the scFv transgenes in the anti-DNP and anti-FITC clones may give rise to variants of surface scFvs with novel binding characteristics. As a first test for how robustly this diversification of the scFvs permitted selection of desired antibody specificities, we asked whether we could converge the specificities of our two starting scFvs (mouse anti-DNP and human anti-FITC) to recognize a common antigen, casein.

**Fig. 2:**
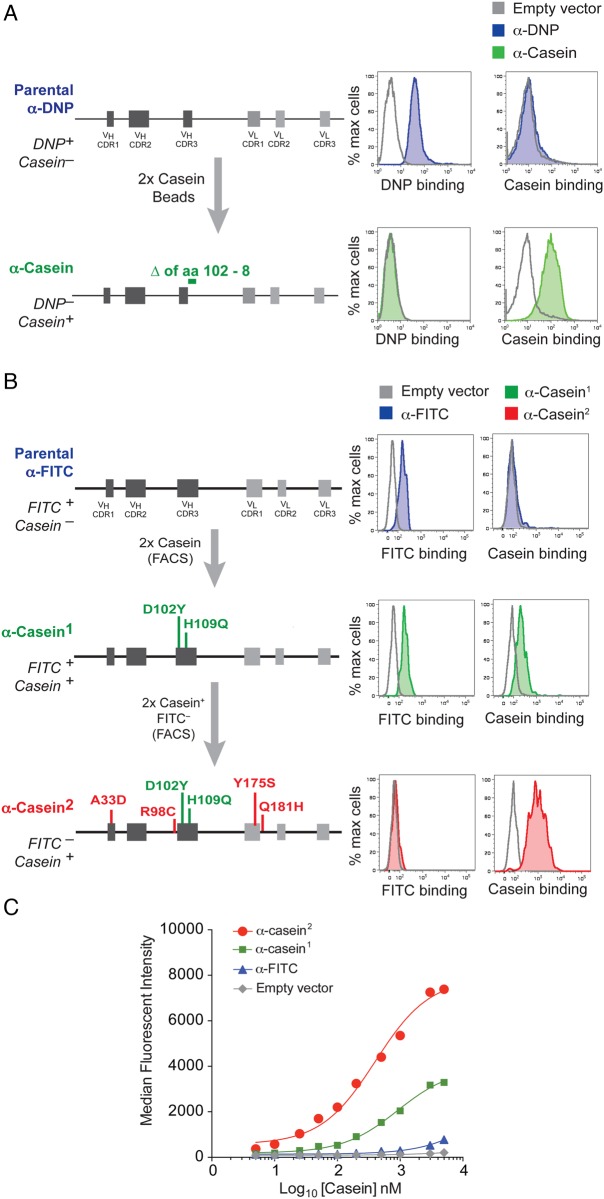
Convergent evolution of mouse anti-DNP and human anti-FITC scFvs to bind a common antigen, casein. (**A**) Summary of the selection steps and isolated mutations for the anti-DNP-to-anti-casein evolution. Mutations determining the evolved antigenic specificity are shown. The flow cytometry histograms to the right show binding of cells transfected with the indicated scFvs to the specified antigens. (**B**) Selection and affinity maturation of an anti-casein scFv from a parental anti-FITC scFv. Anti-casein^1^ is a bispecific scFv that bound FITC and casein, whereas anti-casein^2^ is a monospecific scFv that bound only casein. (**C**) Improved affinity of the anti-casein scFv. Transfected U-2OS cells expressing the indicated scFvs were incubated with increasing concentrations of casein, and the median fluorescent intensity at each concentration was determined by flow cytometry. *K*_D_ was defined as the casein concentration at half maximal binding. From the curve, *K*_D_ of anti-casein^1^ and anti-casein^2^ were determined to be 911 and 402 nm, respectively.

Starting with cells expressing the anti-DNP scFv, we selected for casein binding by performing two rounds of selection with casein-coupled paramagnetic beads, growing up the recovered cells for 7–10 days between selections. This resulted in the isolation of a population that bound casein (Supplementary Fig. S2A). To determine the scFv sequence that conferred this binding phenotype, genomic DNA was extracted from these cells and the scFv-coding region PCR-amplified and sequenced. This revealed a common set of mutations in all independent sequences (Δ102–108 in CDR3 of *V*_H_), which are likely to reflect the changes conferring the new binding specificity, accompanied by random passenger mutations (Supplementary Fig. S2B). To confirm that the identified deletion in *V*_H_ CDR3 conferred the casein-binding phenotype, an scFv-coding sequence harbouring only this deletion was fused to a HEL–TM surface expression cassette and expressed in the human embryonic kidney cell line HEK293T. Clones expressing the scFv/HEL construct were identified by staining for HEL. As expected, cells expressing the mutated scFv (*V*_H_ Δ102–108) but not parental anti-DNP scFv bound casein (Fig. 2A). The selected anti-casein scFv had also lost binding to DNP.

Similarly, we were able to select casein-binding cells from the anti-FITC starting population after two rounds of enrichment with flow cytometry, each time gating for the top 1% of casein-binding cells and again expanding the cells for 7–10 days between selections. As before, the surface scFv of the recovered cells was sequenced and only the coding sequence that carried the common mutations in all sequences was transfected into U-2OS cells. The first scFv derived by this approach, anti-casein^1^, had acquired two point mutations in the *V*_H_ CDR3, but interestingly still retained binding to FITC (Fig. 2B). We therefore performed two further rounds of selection using a more stringent selection regime in which only the brightest 0.5% cells that bound casein but not FITC were progressively enriched (Fig. 2B). This resulted in a new anti-casein^2^ scFv with improved casein binding (*K*_D_ of 402 vs. 911 nm) (Fig. 2C) but that had lost FITC binding.

**Fig. 3:**
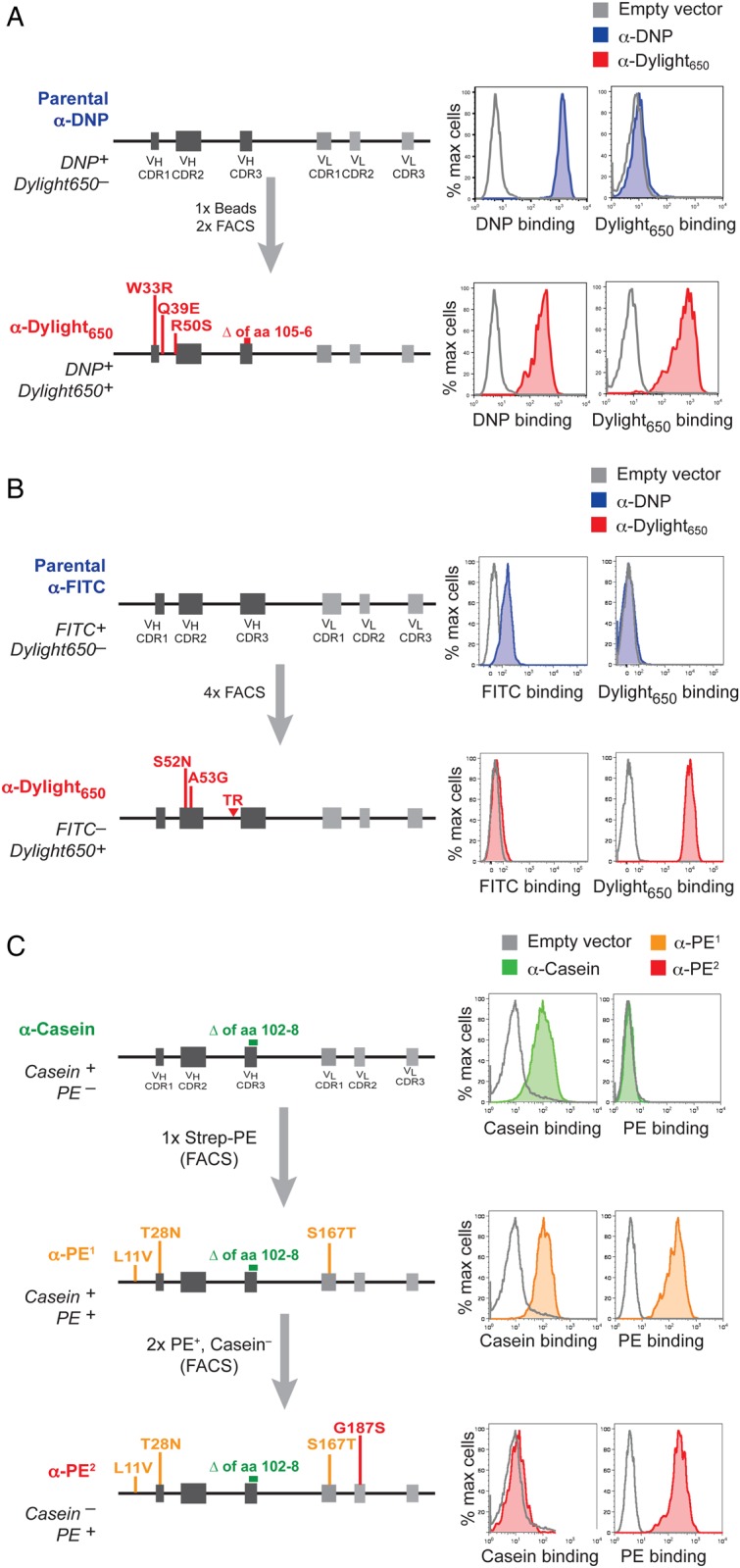
Further scFv selection and evolution examples. (**A**) Derivation of an anti-Dylight_650_ scFv from an anti-DNP scFv parent. (**B**) Derivation of an anti-Dylight_650_ scFv from an anti-FITC scFv parent. Inverted triangle indicates an insertion. (**C**) Further evolution of the anti-casein scFv to a monospecific anti-PE scFv.

We obtained similar success in convergently evolving these anti-DNP and anti-FITC scFvs to KLH conjugated to Dylight_650_ (KLH-Dylight_650_). We evolved the anti-DNP scFv to bind KLH-Dylight_650_ in three rounds of selection and the anti-FITC scFv in four rounds (Fig. 3A and B). Interestingly, in both cases, the evolved scFvs were specific for Dylight_650_ rather than KLH, perhaps reflecting the fact that the starting scFvs are already biased towards binding haptens.

### Rapid evolution of scFv antigen specificities with limited selection steps

To examine the robustness of the system, we next asked whether we could further evolve and alter the specificity of the anti-casein-binding population derived originally from the anti-DNP scFv. We thus selected cells for binding to a streptavidin–phycoerythrin conjugate (strep–PE). One round of flow cytometry selection with strep-PE resulted in the isolation of a first generation anti-PE^1^ scFv that now bound PE as well as casein and that had acquired two further point mutations in *V*_H_ and one in the *V*_L_ (Fig. 3C). We then sought to completely switch the specificity of the scFv so that it only recognized PE. We therefore again adopted a counterselection strategy by selecting for cells binding strep-PE that had lost casein binding. After five rounds, a population carrying a single additional light chain mutation in CDR2 of *V*_L_ was isolated that bound PE, but not casein (anti-PE^2^, Fig. 3C). The system thus can allow facile selection and evolution of novel scFv antigen-binding specificities within only a few rounds.

### Production of soluble IgG1 human antibodies from evolved scFvs

**Fig. 4:**
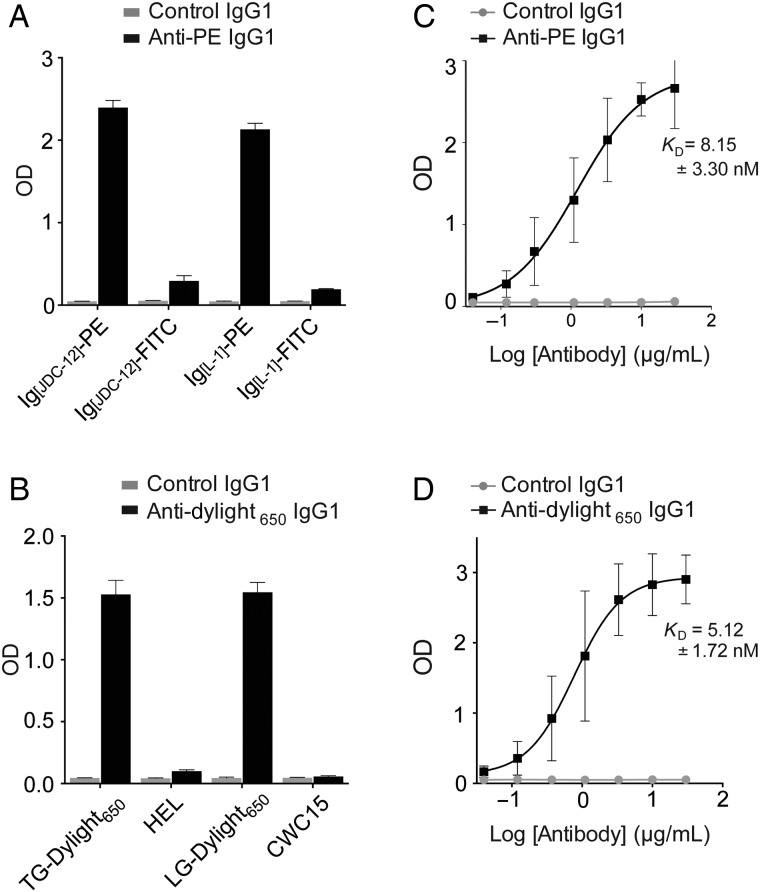
Selected scFvs can be made into full-length IgG1 antibodies. (**A**) Following conversion into a chimeric human/mouse IgG1, the anti-PE^2^ scFv bound immobilized PE-conjugated antigens (two antibodies, clones JDC-12 and L-1) in an ELISA assay but did not bind FITC conjugates of the same antigens. (**B**) Following conversion into a fully human IgG1, the anti-Dylight_650_ scFv (derived from the anti-FITC) bound Dylight_650_-conjugated antigens only (TG, thyroglobulin; HEL, hen egg lysozyme; LG, lactoglobulin; CWC15 is a human spliceosomal factor). (**C**) *K*_D_ determination of the anti-PE^2^ IgG1 by ELISA. (**D**) *K*_D_ determination of the anti-Dylight_650_ IgG1 by ELISA. In all figures, error bars represent 1 SD. Data were obtained from three independent experiments, each with two technical replicates.

To ensure that the antigen specificities we evolved were specific to the heavy and light chain mutations in the scFv, we converted the mouse anti-PE^2^ scFv into a chimeric human/mouse IgG1 and the human anti-Dylight_650_ scFv that was derived from the anti-FITC scFv to a fully humanized IgG1 antibody. To do this, we cloned the heavy and light chain V genes from each scFv into the human Igγ1 and Igκ expression vectors (Tiller *et al.*, 2008) then expressed and purified recombinant IgG1 antibodies from HEK 293T cells. The chimeric anti-PE^2^ and human anti-Dylight_650_ IgG1 antibodies produced this way retained binding to PE and Dylight_650_, respectively (Fig. 4A and B), indicating that binding to these antigens was indeed conferred by the V genes. In an ELISA assay, the anti-PE^2^ IgG1 bound Strep-PE with a *K*_D_ of 8.15 ± 3.30 nm whereas the anti-Dylight_650_ IgG1 bound Strep-Dylight_650_ with a *K*_D_ of 5.12 ± 1.72 nm (Fig. 4C and D).

### Creation of a diverse mutating human scFv library

Starting from a single scFv is likely to allow access to only a limited gamut of epitopes. Indeed, our attempts at selecting for scFvs that bind three other test antigens (thyroglobulin, ubiquitin and human IgG) using the anti-FITC clone did not yield any binding scFvs after five rounds of selection. This may be due to a limit to the number of unique, functional scFv variants that can be generated based on only a single scFv framework. To improve this, we started building a larger scFv library by creating further DT40 clones expressing unique scFvs. Seven lines were created using random scFvs amplified from the Tomlinson synthetic scFv library (de Wildt *et al.*, 2000): Tomlinson 812, 813, 815, 817, 840, 842 and 843 (Supplementary Fig. S3). The scFvs in this library are derived from a single human antibody framework, where the heavy chain is made up of V3-23/DP-47 and J_H_4b genes and the κ light chain made up of the O12/O2/DPK9 and Jκ1 genes. This library has been previously used for the successful isolation of a range of antigen-specific scFvs (Robert *et al.*, 2006; Boulter-Bitzer *et al.*, 2009). In a parallel approach, CDR3s from a set of human antibodies obtained from humanized translocus mice (Pruzina *et al.*, 2011) were grafted in place of the *V*_H_-CDR3 of the initial anti-FITC scFv. From this, we generated three more DT40 scFv knock-ins—FITC 832, 833 and 1039 (Supplementary Fig. S3)—making a final library of 11 unique DT40 lines.

**Fig. 5:**
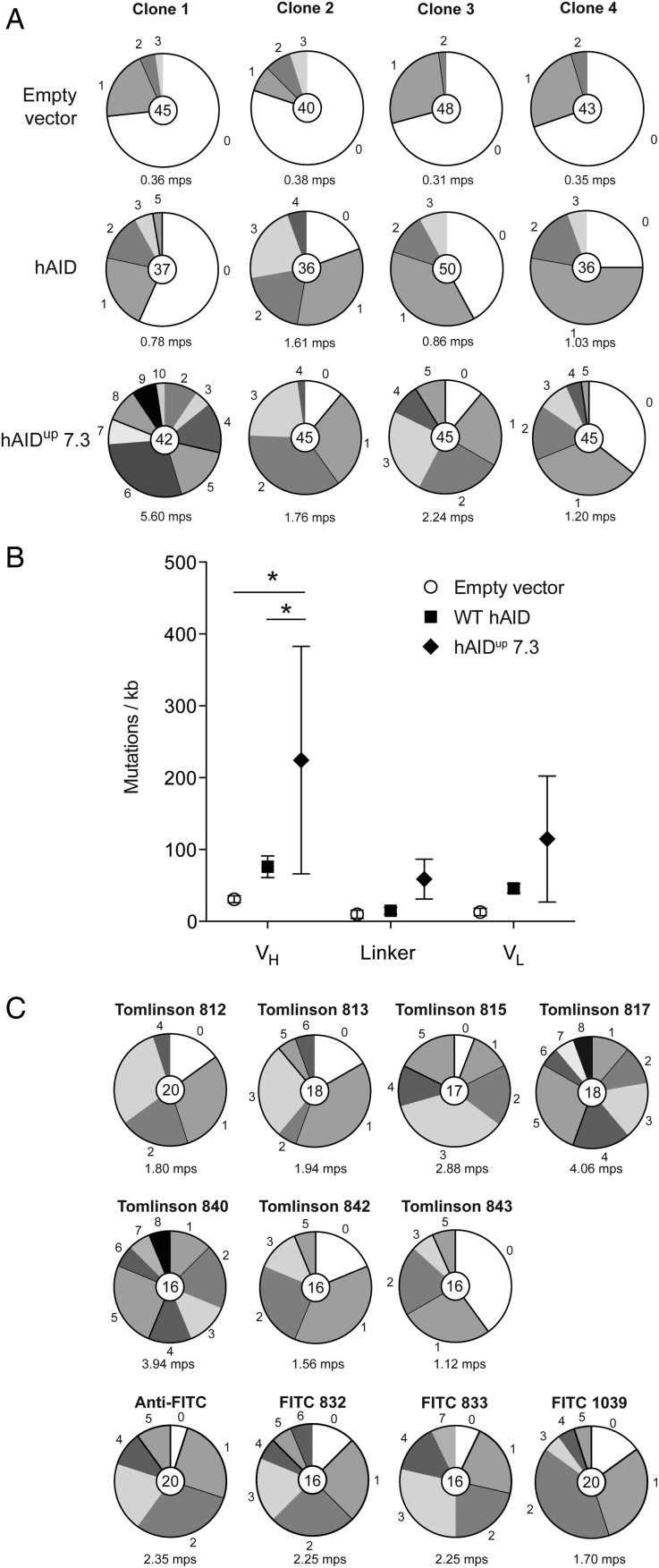
Construction of a mini scFv library and enhancing scFv diversification. (**A**) Mutation load in the scFv transgene of four Tomlinson 817 clones overexpressing different hAID variants (see also Supplementary Fig. S4). The number in the middle of each pie chart indicates the total number of sequences analysed, whereas the numbers in the periphery of the different segments indicate the number of mutations per sequence. Segment sizes are proportional to the number of sequences carrying the indicated numbers of mutations per sequence. The number below each chart indicates the average number of mutations per sequence (mps). (**B**) Mutation load in the *V*_H_, linker and *V*_L_ segments of the scFv following overexpression of the different hAID variants. Each point represents the average number of mutations per kb in that segment of the four clones overexpressing the same hAID variant. The whiskers represent 1 SD. (**C**) Mutation load in the scFv transgene of the 11 library clones overexpressing hAID^up^ 7.3.

Although we had created a larger scFv library, a further limit to accessing the widest possible range of epitopes was the mutation rate. Although robust, the mutation rate generated by endogenous AID in DT40 can be accelerated by expression of both increased amounts of AID (Arakawa *et al.*, 2004) and by expressing hyperactive AID derivatives (Wang *et al.*, 2009). To test this, we first overexpressed FLAG-tagged wild-type human AID (hAID) in cells expressing the Tomlinson 817 scFv and assessed mutation accumulation over 4 weeks. Under these conditions, endogenous chicken AID generated 0.35 ± 0.01 mutations per sequence (Fig. 5A). Overexpression of hAID increased this to 1.07 ± 0.19 mutations per sequence. This was further increased to 2.70 ± 0.99 mutations per sequence by the expression of a human AID variant, hAID^up^ 7.3 (Wang *et al.*, 2009), which exhibits increased catalytic activity. Overexpression of this variant not only increased the total number of mutations per sequence but also increased the mutation load in the *V*_L_ segment, which is otherwise mutated at a lower level than the *V*_H_ segment due to its greater distance from the promoter (Fig. 5B). Interestingly, expression of hAID^up^ 7.3 was more variable than wild-type hAID but nevertheless was able to induce high levels of mutation (Supplementary Fig. S4). As hAID^up^ 7.3 is more mutagenic, we overexpressed this mutant in all of our 11 library clones. In each, hAID^up^ 7.3 induced robustly diverse mutagenesis of the scFv transgenes (Fig. 5C).

### Selection from the human scFv library

To ascertain the utility of this library, we performed scFv selections using three test antigens: the human splicing factor CWC15 (hCWC15), a very highly conserved mammalian protein, mouse interleukin 33 (mIL-33) and human interferon gamma (hIFNγ), the latter a possible target for human therapy. To avoid competition between scFv-expressing DT40 clones, we expanded each of the 11 library clones independently, only combining them before the first selection step.

**Fig. 6:**
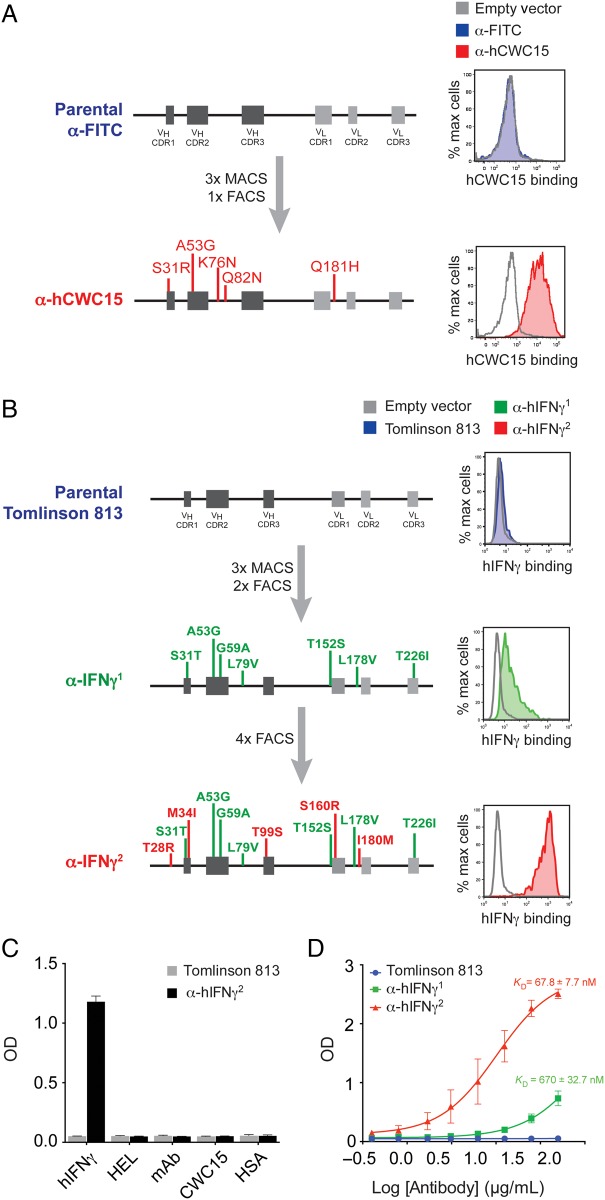
Selection of scFvs of desired specificities from the DT40 scFv mini-library. (**A**) Isolation of an anti-hCWC15 scFv. Sequence analysis revealed that this scFv was derived from the parental anti-FITC (Supplementary Fig. S3). (**B**) Selection and affinity maturation of an scFv against human IFNγ. Sequence analysis revealed that the parental scFv was Tomlinson 813 (Supplementary Fig. S3). (**C**) The anti-hIFNγ^2^ scFv was recombinantly expressed as a human scFv-Fc. The resulting antibody retained specific binding to immobilized IFNγ in an ELISA assay but did not bind other antigens (hIFNγ, human interferon gamma; HEL, hen egg lysozyme; mAb, mouse IgG; CWC15 is a human spliceosomal factor; HSA, human serum albumin). (**D**) Recombinantly expressed anti-IFNγ^1^ and hIFNγ^2^ scFv-Fcs were titrated, and binding to hIFNγ was determined at each concentration by ELISA. From the curve, *K*_D_ of the anti-IFNγ^1^ and IFNγ^2^ scFv-Fcs were determined as 670 ± 32.65 and 67.80 ± 7.69 nm, respectively. In C and D, error bars represent 1 SD. Data were obtained from three independent experiments, each with two technical replicates.

While selection with mIL-33 did not yield any binders, we obtained cells that bound hCWC15 after three rounds of enrichment using paramagnetic beads plus one further round with FACS (Fig. 6A). Sequencing analysis revealed that this scFv was derived from an anti-FITC scFv framework and contained five independent mutations, four in *V*_H_ and one in *V*_L_. We obtained similar success with hIFNγ. Three rounds of enrichment using paramagnetic beads and two further rounds by FACS resulted in a heavily mutated scFv carrying seven conserved mutations in both the heavy and light chain of the Tomlinson 813 scFv that was able to bind hIFNγ, but with an apparently quite low affinity (anti-hIFNγ^1^, Fig. 6B). After four further rounds of selection by FACS, a higher affinity population was isolated that carried a further five mutations spread across the heavy and light chains of the scFv (anti-hIFNγ^2^, Fig. 6B).

To enable convenient large-scale purification of this scFv, we recombinantly expressed the anti-hIFNγ scFvs in the format of scFv-Fcs, where the scFv is joined to the human Igγ1 Fc region (Shu *et al.*, 1993). The purified anti-hIFNγ^2^ scFv-Fc retained specific binding to hIFNγ, with minimal binding to other antigens (Fig. 6C), and by ELISA exhibited an apparent *K*_D_ of 67.80 ± 7.69 nm, a 10-fold increase over the initial anti-hIFNγ^1^, which exhibited a *K*_D_ of 670 ± 32.65 nm (Fig. 6D).

## Discussion

Chicken DT40 cells can be used to harness the three key features of antibody production in the humoral immune response: repertoire generation, SHM and affinity selection (Cumbers *et al.*, 2002). Here, we report modification of the cells to allow both *de novo* isolation of desired human antibody binding specificities and the evolution of existing scFvs. The methodology is relatively cheap and straightforward to implement and combines advantageous features of both phage display and transgenic mice. As with other display approaches, hypermutating cell lines such as DT40 eliminate the need for animal immunization and subsequent hybridoma production. It also bypasses restrictions imposed by *in vivo* tolerance mechanisms that can limit the production of high affinity antibodies against conserved proteins following immunization.

As well as isolation of novel specificities, a particular utility of the system is the ease with which it can be used to refine the affinity and specificity of scFvs isolated by other methods. This is facilitated by the simple and efficient single-step targeting of the scFv into the light chain locus of DT40. Furthermore, the approach avoids the repetitive antibody gene isolation, *in vitro* mutagenesis and re-transfection that is required to mimic affinity maturation in non-diversifying display systems (Gram *et al.*, 1992; Chowdhury and Pastan, 1999; Boder *et al.*, 2000; Steidl *et al.*, 2008).

Like phage display, the DT40 system also allows for careful control of the selection strategy. We have employed both flow cytometry and magnetic beads to select binding variants. Both approaches have proved successful, but our sample size is not sufficiently large to be able to come down in favour of one approach over the other. Nevertheless, we have generally adopted a magnetic beads-first-flow cytometry-later strategy when selecting for binders from our mini-library as it is possible to rapidly screen large numbers of cells with magnetic beads carrying a dense array of antigen on their surface to isolate initial low affinity binders.

The on-going diversification of the scFvs in DT40, which is coupled to their surface display, allows the use of a relatively small starting repertoire of V genes. We have not systematically addressed the question of the optimal size of the starting scFv library needed to allow the initial selection of scFv variants against the majority of chosen antigens. However, our library contained just 11 unique scFv fragments, and from this, we were successful in isolating antibodies against 2 out of 3 test antigens. Indeed, even starting from single scFvs resulted in a substantial ‘hit’ rate. Surface display libraries often comprise 10^7^ to 10^11^ unique variants (Ponsel *et al.*, 2011). Given that the rapid growth of DT40 cells and the mutation loads we report, generation of 10^7^ to 10^8^ variants from a single starting scFv is readily achievable. Thus, in future, a carefully selected starting scFv library of under 100 scFvs should result in final library sizes approaching those in phage display.

## Supplementary data

Supplementary data is available at *PEDS* online.

## Funding

The work was supported by the Medical Research Council (U1051178808 and U105178806). Funding to pay the Open Access publication charges for this article was provided by The Medical Research Council.

## Supplementary Material

Supplementary Data
