# Supplementary material for: Directed evolution of human scFvs in DT40 cells
Source: Protein Eng Des Sel. 2015 Oct 30;29(2):39–48. doi: 10.1093/protein/gzv058 (PMC4840456; doi:10.1093/protein/gzv058)
Supplement: Supplementary Data [file supp_29_2_39__index.html]

Directed evolution of human scFvs in DT40 cells — Directed evolution of human scFvs in DT40 cells — Supplementary Data 

# Directed evolution of human scFvs in DT40 cells

## Supplementary Data

Supplementary Data

- Supplementary Data - Docx file
