# Supplementary material for: Directed evolution of human scFvs in DT40 cells
Source: Protein Eng Des Sel. 2015 Oct 30;29(2):39–48. doi: 10.1093/protein/gzv058 (PMC4840456; doi:10.1093/protein/gzv058)
Supplement: Supplementary Data [file supp_gzv058_gzv058supp.docx]

**Directed evolution of human scFvs in DT40 cells**

Alfred W.Y. Lim^*^, Gareth T. Williams^*^, Cristina Rada and Julian E. Sale^†^.

Medical Research Council Laboratory of Molecular Biology, Francis Crick Avenue, Cambridge, CB2 0QH, UK.

**Supplementary Figures**

**Figure S1**

**
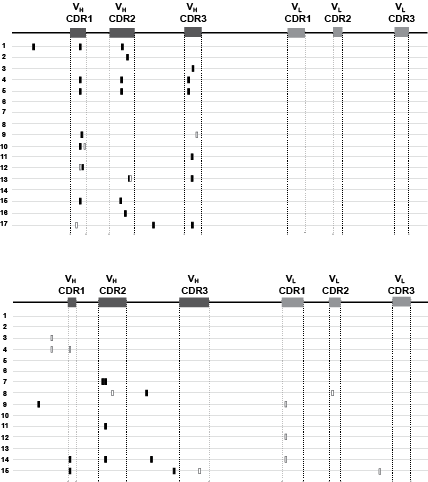
**

**Supplementary Figure 1.** Distribution of point mutations along the anti-DNP scFv (top panel) and anti-FITC scFv (bottom panel) transgenes in unselected populations of targeted clones. Each horizontal line represents an independent sequence. Regions corresponding to the CDRs are indicated. Hollow boxes represent transition mutations while filled boxes represent transversion mutations.

**Figure S2**

**
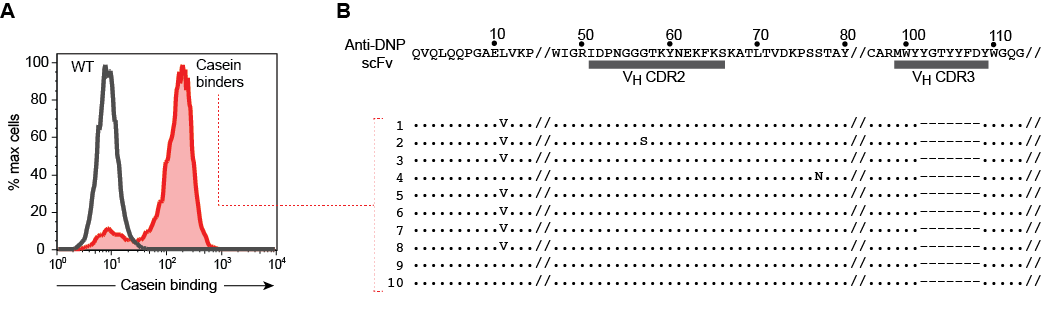
**

**Supplementary Figure 2.** A. Flow cytometry histogram depicting casein binding by wild type DT40 and cells that are recovered after two successive rounds of selection with casein-conjugated paramagnetic beads. B. Sequence analysis of the scFv in the population of casein-binding cells. Residues contributing to CDRs in the parental anti-DNP scFv are indicated. Each horizontal line represents an independent sequence. Dots represent sequence identity with the parental scFv sequence and a hyphen represents an amino acid deletion. In this example, the common feature in all clones is a deletion of amino acids 102 – 8.

**Figure S3**

**
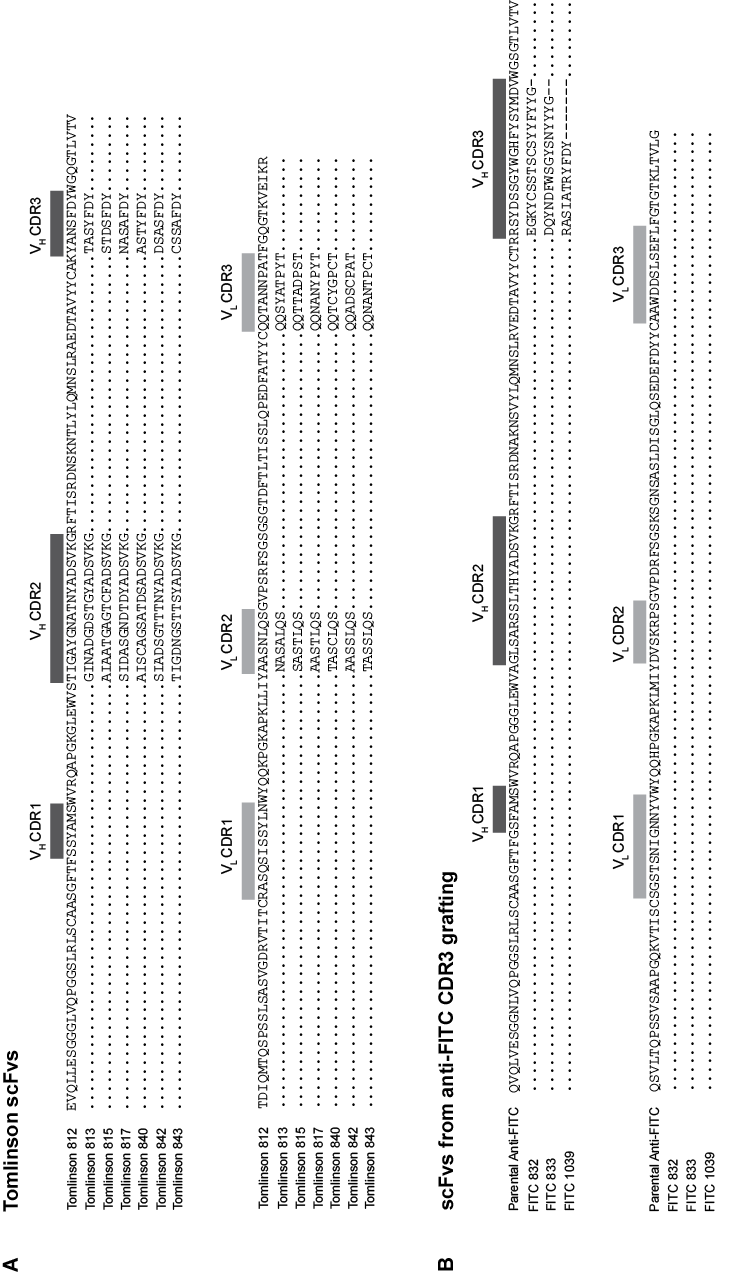
**

**Supplementary Figure 3. A.** Sequence alignment of the seven different synthetic Tomlinson scFvs depicting their variations in the CDR2s and 3s of V_H_ and V_L_. B. Sequence alignment of the human anti-FITC scFv with the three new antibodies generated by CDR3 grafting onto the former’s framework. Dots indicate sequence identity, hyphen indicates deletion.

**
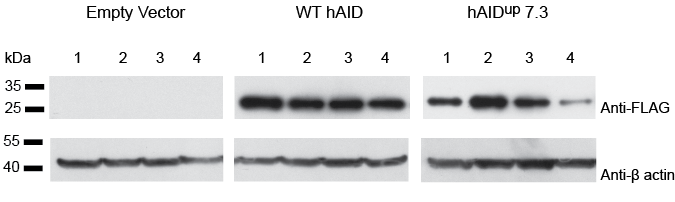
Figure S4**

**Supplementary Figure 4.** Relative abundance of FLAG-tagged wild type human AID (hAID) and hAID^up^ 7.3 in four independent Tomlinson 817 clones four weeks post-transfection. Detection of hAID was performed using an anti-FLAG antibody and the blots were re-probed with an anti-β-actin antibody to control for loading.
